# Intrinsic Photoluminescence of Solid-State Gold Nanoclusters: Towards Fluorescence Lifetime Imaging of Tissue-Like Phantoms Under Two-Photon Near-Infrared Excitation

**DOI:** 10.3389/fchem.2021.761711

**Published:** 2021-10-21

**Authors:** Alexandru-Milentie Hada, Ana-Maria Craciun, Simion Astilean

**Affiliations:** ^1^ Nanobiophotonics and Laser Microspectroscopy Center, Interdisciplinary Research Institute in Bio-Nano-Sciences, Babes-Bolyai University, Cluj-Napoca, Romania; ^2^ Faculty of Physics, Babes-Bolyai University, Cluj-Napoca, Romania

**Keywords:** gold nanoclusters, intrinsic photoluminescence, solid state, tissue phantom, FLIM, two-photon excitation

## Abstract

Gold nanoclusters (AuNCs) have attracted extensive attention as light-emissive materials with unique advantages such as high photostability, large Stoke shifts and low toxicity. However, a better understanding of their solid-state photoluminescence properties is still needed. Herein, we investigated for the first time the intrinsic photoluminescence properties of lyophilized bovine serum albumin stabilized AuNCs (BSA-AuNCs) *via* fluorescence lifetime imaging microscopy (FLIM) studies performed under both one and two photon excitations (OPE and TPE) on individual microflakes, combined with fluorescence spectroscopic investigations. Both in solution and solid-state, the synthesized BSA-AuNCs exhibit photoluminescence in the first biological window with an absolute quantum yield of 6% and high photostability under continuous irradiation. Moreover, under both OPE and TPE conditions, solid BSA-AuNCs samples exhibited a low degree of photobleaching, while FLIM assays prove the homogeneous distribution of the photoluminescence signal inside the microflakes. Finally, we demonstrate the ability of BSA-AuNCs to perform as reliable bright and photostable contrast agents for the visualization of cancer tissue mimicking agarose-phantoms using FLIM approach under non-invasive TPE. Therefore, our results emphasize the great potential of the as synthesized BSA-AuNCs for *ex vivo* and *in vivo* non-invasive NIR imaging applications.

**GRAPHICAL ABSTRACT:**
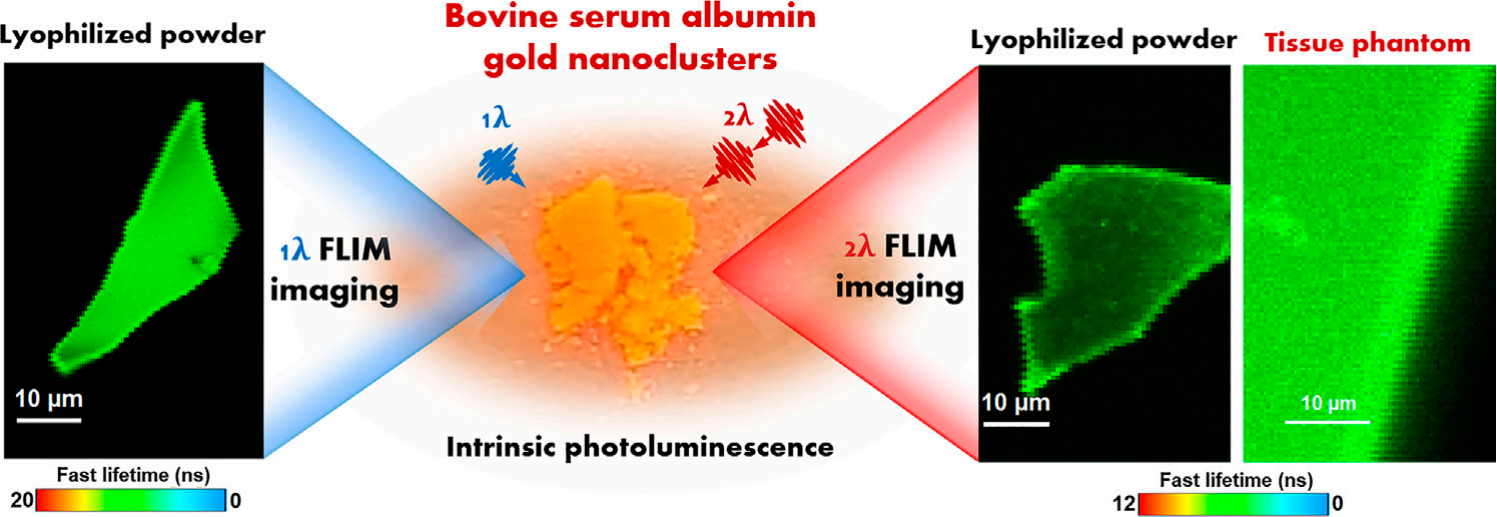


## Introduction

Metal nanoclusters (NCs) are a class of extremely small and versatile nanomaterials (1–5 nm) exhibiting unique chemical and optical properties. They are composed of a few and up to 200 atoms, having a size comparable to the Fermi wavelength, and are protected by an organic molecular ligand enriching them with molecular-like properties such as large Stoke shift and tunable intrinsic photoluminescence (PL) (Akyüz et al., 2020), similar to upconversion nanocrystals (Wang et al., 2021a; Wang et al., 2021b). Owing to the aforementioned characteristics, NCs show potential in various applications like cell and tissue imaging (Liu et al., 2011; Chen et al., 2016), biolabeling (Xu et al., 2020), catalysis (Li and Jin, 2013), molecular identification (Zhang et al., 2018), ion detection (Ding et al., 2014; Hada et al., 2021c), etc. Among the most common metal NCs (gold, silver, copper), gold nanoclusters (AuNCs) present noteworthy advantages in terms of biocompatibility and photostability (Zuber et al., 2019). Therefore, lately, the research interest was focused mainly on improving the PL properties and photostability of AuNCs.

AuNCs can be synthesized using a wide range of capping agents such as polymers (Tsunoyama et al., 2005), amino acids (Liu et al., 2017), peptides (Fabris et al., 2006), proteins (Xie et al., 2009; Hada et al., 2021c), etc. Amongst, a very common and easy to obtain protein, bovine serum albumin (BSA), is extremely used in the synthesis of AuNCs due to its appealing properties such as small dimensions, stability and biocompatibility (Porret et al., 2020). The first synthesis of AuNCs wrapped in a BSA corona (BSA-AuNCs), exhibiting an intense far-red emission, was reported by Xie et al. (2009). Later, multiple studies reported on the dependence of colloidal BSA-AuNCs PL on temperature, pH, time, etc. (Chen et al., 2014; Zhang et al., 2014; Chib et al., 2015). However, there is a lack of studies on the properties of AuNCs, particularly BSA-AuNCs, in solid-state. Such studies could ensure a better understanding of the PL process occurring in AuNCs inside complex heterogeneous cellular environment, when exploited in biomedical applications.

A powerful technique useful for the investigation of photoluminescent nanomaterials is fluorescence lifetime imaging microscopy (FLIM), owing to enhanced sensibility and contrast compared to the conventional steady-state fluorescence imaging. The surpassing imaging quality is gained by mapping the fluorescence lifetime, a parameter that can detect even the smallest structural and environmental changes (Orte et al., 2013; Craciun et al., 2021). In our previous work (Hada et al., 2021b), we have already demonstrated that BSA-AuNCs can perform as valuable *in vitro* fluorescence contrast agents for FLIM-based cancer cell imaging, under one-photon excitation (OPE). However, when it comes to medical applications, imaging techniques based on two-photon excitation (TPE) come with very important advantages such as improved sensitivity and axial resolution, reduced photobleaching, higher penetration depth and lack of photodamage to living cells due to non-invasive near-infrared (NIR) excitation, which eliminates out-of-focus absorption, providing background-free information, while maintaining a high image resolution (Benninger and Piston, 2013). Despite the fact that the PL of AuNCs in solution under two-photon excitation (TPE) has already been reported in a bunch of studies (Bertorelle et al., 2018; Vangara et al., 2018; Olesiak-Banska et al., 2019), up to our knowledge, the combined TPE-FLIM method was not previously used to investigate the PL properties of AuNCs in solid-state or inserted in tissue-like phantoms.

Therefore,in this work, we investigate, for the first time, the intrinsic PL of lyophilized BSA-stabilized AuNCs and demonstrate the persistence of OPE and TPE PL properties in solid samples. Moreover, we demonstrate the ability of as-lyophilized BSA-stabilized AuNCs to be inserted in solid tissue-like phantoms and operate as photostable and bright FLIM contrast agent under TPE, which is relevant for future *ex vivo* and *in vivo* imaging applications. Notably, we demonstrated the consistency of BSA-stabilized AuNCs optical properties both in solution and solid-state (lyophilized powder) as well as their high photostability under continuous irradiation in the first biological window. The absolute quantum yield of the synthesized colloidal BSA-AuNCs was calculated to be 6%. Furthermore, under both OPE and TPE conditions, 99.5% of the initial PL of BSA-AuNCs in solid-state, was recovered after the irradiation time, pointing towards a high photostability and low degree of photobleaching. The performed FLIM assays demonstrate homogeneous PL signal inside individual powder microflakes while fluorescence lifetime decay analysis revealed the presence of two PL lifetime components assigned to the specific intersystem crossing (ISC) and prompt fluorescence emission (PF) transitions occurring between the energy levels of BSA-AuNCs. Moreover, the effect of laser excitation power on both OPE and TPE PL of BSA-AuNCs microflakes was successfully evaluated *via* FLIM. The final aim of our study was to provide the first demonstration of using BSA-AuNCs as fluorescence contrast in a simulated *ex vivo* environment (i.e., agarose tissue-like phantom) using the TPE-FLIM method, as a novel proof of concept for AuNCs-based NIR tissue imaging approach. TPE-FLIM results proves that BSA-AuNCs perform as efficient contrast agents when embedded in a cancer tissue-mimicking solid phantom while maintaining their PL spectral and temporal characteristics. Considering that tissue-like phantoms play a significant role in the development and validation of new bioimaging technologies, our results demonstrate that BSA-AuNCs show great potential for future *ex vivo* and *in vivo* imaging applications.

## Materials and Methods

### Chemicals

Bovineserum albumin (BSA, heat shock fraction, pH 7, ≥98%), hydrogen tetrachloroaurate (III) trihydrate (HAuCl_4_·3H_2_O), agarose high resolution, tris buffered saline (TBS), hemoglobin from bovine blood (suitable for protease substrates, substrate powder) and intralipid (20%, emulsion, phospholipid stabilized soybean oil) were procured from Sigma-Aldrich, while the sodium hydroxide and ascorbic acid were purchased from Merck. All reagents used were of analytical grade. The solutions were prepared using ultrapure water obtained with a purification system (Milli-Q, Millipore, Merck, Massachusetts, United States), having a resistivity of at least 18 MΩcm.

### Synthesis of Bovine Serum Albumin Stabilized AuNCs

The BSA-AuNCs were obtained through a two-step chemical procedure, using BSA as both reducing and stabilizing agent, as previously described in our work (Hada et al., 2021b).

### Lyophilization of Bovine Serum Albumin Stabilized AuNCs

Prior to the lyophilization process, the purified BSA-AuNCs were kept for 24 h in the freezer. Next, the BSA-AuNCs were lyophilized in a Biobase BK-FD 10 Series Vacuum Freezer pre-cooled at −60°C with a vacuum degree of less than 10 Pa.

### Preparation of Agarose-Phantom with Bovine Serum Albumin Stabilized AuNCs

For the synthesis of Agarose-Phantom@BSA-AuNCs, we adapted and optimized a previously reported procedure (Pleijhuis et al., 2014). Briefly, in order to obtain the agarose-phantom mixture, 0.25 g of bovine hemoglobin were dissolved in 15 ml of TBS under vigorous magnetic stirring at room temperature. After 15 min, 0.2 ml of intralipid (20%) and 0.75 g of agarose were added to the hemoglobin solution and the temperature was raised to 95°C, while the mixture was left under magnetic stirring until full dissolution of the agarose. Next, 0.6 ml of the purified BSA-AuNCs was added to 1.4 ml of still hot previously obtained agarose-phantom mixture and left to solidify at room temperature for 1 h. The Agarose-Phantom@BSA-AuNCs was stored at 4°C for later use. Agarose mimics the texture of real tissue, while intralipid and hemoglobin were added to simulate the scattering and the absorption of real cancer-tissue, respectively.

### Equipment

The extinction spectra were recorded using a 1 nm spectral resolution JASCO V-670 UV-Vis-NIR spectrophotometer (Tokyo, Japan) with a 2 mm Helma quartz cuvette. The analysis was performed using the JASCO Spectra Manager program.

The steady-state fluorescence spectra were obtained with a JASCO FP6500 spectrofluorometer (Tokyo, Japan) having a spectral resolution of 1 nm and equipped with a xenon excitation lamp (150 W). The excitation and emission band width were set at 3 nm, while the excitation wavelength was fixed at 405 nm for both the colloidal solution and lyophilized powder of BSA-AuNCs. The steady-state fluorescence measurements in solution were registered in a 5 × 5 Helma quartz cuvette, while the powder measurements were acquired using the epifluorescence module (EFA 383). The spectra were analyzed with the JASCO Spectra Manager program. The absolute quantum yield was measured under an excitation of 405 nm using an integrating sphere accessory (ILF-835) of the JASCO FP-8600 spectrofluorometer in a 3 mm thickness quartz cuvette. The internal quantum yield (QY) was calculated using the JASCO spectra manager software, based on the following equation

$$QY=\frac{E_{2}}{L_{1}-L_{2}},$$ (1)

where E_2_ – the area under the PL emission band, L_1_ – the area under the excitation band, L_2_ – the area under the unabsorbed excitation band.

Fluorescence lifetime decay curves, intensity time-traces and confocal FLIM images of the BSA-AuNCs powder and the Agarose-Phantom@BSA-AuNCs were obtained with a time-resolved confocal fluorescence MicroTime200 microscope system (PicoQuant, Berlin, Germany) equipped with an Olympus IX 71 microscope. The OPE fluorescence measurements were performed under 405 nm excitation laser (diode laser LDH-D-C-405, 40MHz, 0.07–1 μW at the probe site, PicoQuant), while the TPE fluorescence measurements were executed under 810 nm excitation laser (Mira 900 Titanium Saphire (Ti-Sa) tunable femtosecond laser, 76 MHz, 5–20 mW at the laser cavity exit site). Both OPE and TPE signals were collected through a UPLSAPO 60×/NA 1.2 water immersion objective and spectrally filtered by a FF01-519/LP emission filter (Semrock, United States). For TPE, the FF01-750/SP (Semrock, United States) filter was additionally used. Afterwards, the signal was focused on a PDM Single Photon Avalanche Diode (MPD) and processed by a PicoHarp 300 Time-Correlated Single Photon Counting (TCSPC) data acquisition unit. The piezo x-y scanning table and the PoFcz-piezo actuator attached to the microscope assure the system capacity to perform imaging assays. OPE and TPE time traces and decay curves were registered from different points of interest from the OPE/TPE FLIM images. The spectral information from the selected points of the Agarose-Phantom@BSA-AuNCs was recorded with a SR-163 spectrograph equipped with an Andor Technology camera (Newton 970EMCCD), which is connected through a 50 μm optical fiber to the MicroTime200 main optical unit. The TPE PL spectrum was obtained using an integration time of 20 s. The fluorescence lifetime values were obtained by tailfit fitting operations until χ^2^, standard deviation and residuals reached optimal values.

Transmission electron microscopy (TEM) images of BSA-AuNCs were obtained using an ultra-high resolution Hitachi microscope (HD2700).

## Results and Discussion

### Characterization of Bovine Serum Albumin Stabilized AuNCs

**FIGURE 1:**
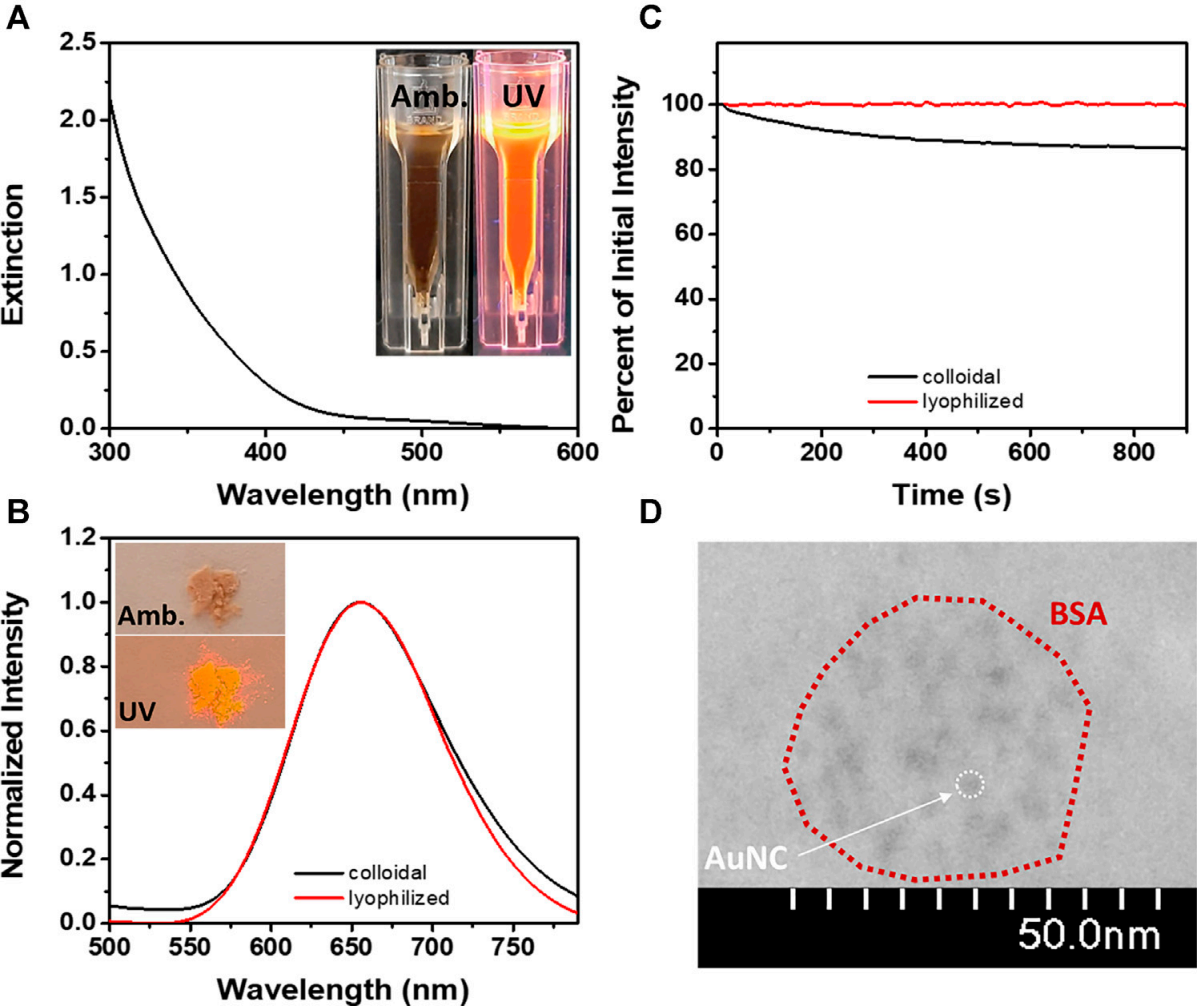
**(A)** The extinction spectrum of BSA-AuNCs (inset – images of BSA-AuNCs in aqueous solution under amb. and UV light). **(B)** The emission spectra of BSA-AuNCs in solution (black line) and as lyophilized powder (red line) at an excitation of 405 nm (inset – images of BSA-AuNCs as lyophilized powder under amb. and UV light). **(C)** The photostability of BSA-AuNCs in solution (black line) and as lyophilized powder (red line) under continuous excitation at 405 nm for 15 min. **(D)** HR-TEM image of the BSA-AuNCs.

The as-prepared BSA-AuNCs exhibit both in aqueous dispersion (inset – Figure 1A) and powder sample (inset – Figure 1B) a light brown color under ambiental (amb.) light excitation, while under UV light excitation turns reddish. This is the first indicator of the intrinsic PL of the obtained BSA-AuNCs. Also, the absence of a localized surface plasmon resonance (LSPR) band in their absorption spectrum (Figure 1A), suggests the formation of cluster-size nanoparticles. This phenomenon (no LSPR) takes place usually when the nanostructure’s dimension approaches the Fermi wavelength of metals (∼2 nm), transforming the continuous energy level band into discrete ones and giving NCs molecule-like properties. In consequence, NCs show intrinsic PL originating from sp-sp (intraband) and sp-d (interband) transitions (Yang et al., 2020). As shown in Figure 1B, BSA-AuNCs exhibit both in aqueous solution and as lyophilized powder a strong emission at 655 nm under 405 nm excitation. This proves their great stability and conservation of fluorescence properties in different states of matter, which is a great desiderate for various fluorescence-based application.

It was previously demonstrated that the BSA-AuNCs are photoluminescent under a large range of excitation wavelengths (Hada et al., 2021b). In this work, we have selected to perform our steady-state fluorescence investigations under continuous 405 nm laser excitation to match the excitation wavelength of the pulsed laser used in our future time-resolved spectroscopy and microscopy experiments. The absolute quantum yield of the BSA-AuNCs in solution under an excitation of 405 nm was calculated to be 6%. Even though the quantum yield of BSA-AuNCs was relatively calculated to be around this value in other works (Xie et al., 2009; Hofmann et al., 2014; Zhang et al., 2017), to our knowledge, this is the first time the absolute value is reported. Besides their intrinsic PL, NCs are also known for their high photostability (Le Guével et al., 2012). As shown in Figure 1C, under continuous excitation at 405 nm for 15 min, the BSA-AuNCs retain in solution 86.8% of their original PL, while the powder sample reaches a value of 99.5%, which represents an extremely important advantage for future bioimaging applications. The morphology and structural characteristics of the BSA-AuNCs were rigorously detailed in our previous work as obtained from TEM, XPS and DLS measurements (Hada et al., 2021b). Briefly, the BSA-AuNCs consists of AuNCs with an average size of 3 nm (Figure 1D) embedded in a BSA corona of 25 ± 12 nm. The aforementioned results prove the successful formation of BSA-AuNCs with an intrinsic far-red PL.

### One and Two Photon Excitation Fluorescence Lifetime Imaging Microscopy Assays on Bovine Serum Albumin Stabilized AuNCs Powder

**FIGURE 2:**
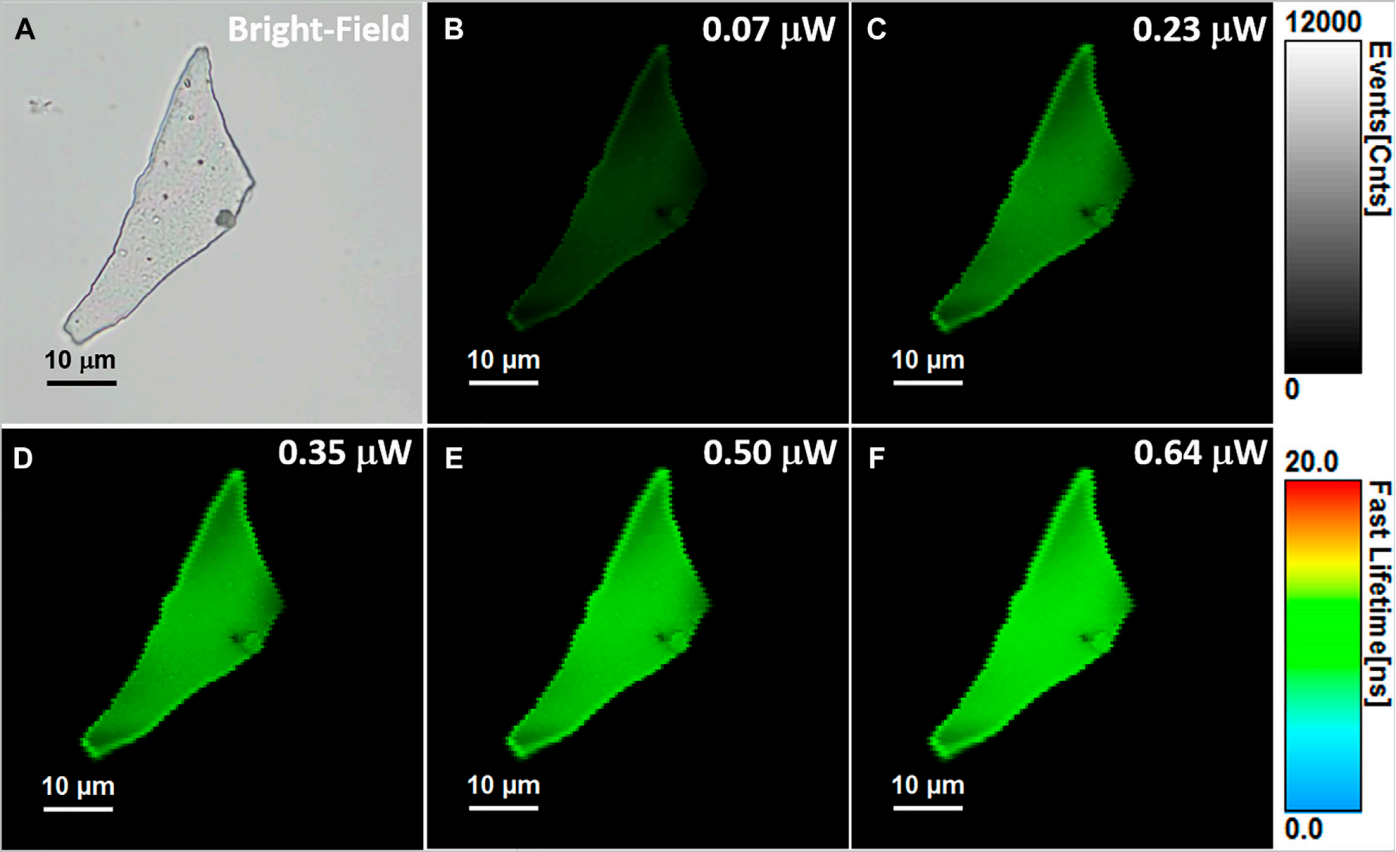
**(A)** Optical image of the investigated BSA-AuNCs microflake powder. **(B–F)** OPE FLIM images of BSA-AuNCs microflake powder under 405 nm excitation using different laser power on the probe, ranging from 0.07 to 0.64 µW. All FLIM images are presented on the same intensity (0-12,000 counts) and lifetime (0–20 ns) scales. The images were recorded at a resolution of 150 × 150 px with an imaging speed of 64 s.

As it was previously demonstrated, FLIM is a very attractive fluorescence microscopy technique, overall superior to conventional fluorescence microscopy due to the enhanced contrast provided by sensitivity of fluorescence lifetime to local environment (Orte et al., 2013). Due to the lack of studies on the PL of the BSA-AuNCs in a solid-state, the next part of the study was focused on the fluorescence lifetime imaging of BSA-AuNCs powder in order to expose the spectral and time-resolved response of the NCs in experimental conditions close to the biological ones. Therefore, we recorded the OPE FLIM images of the same BSA-AuNCs microflake under 405 nm excitation, using different laser powers on the probe ranging between 0.07 and 0.64 µW. The collected OPE FLIM images and the corresponding optical image of the investigated microflake are presented in Figure 2.

**FIGURE 3:**
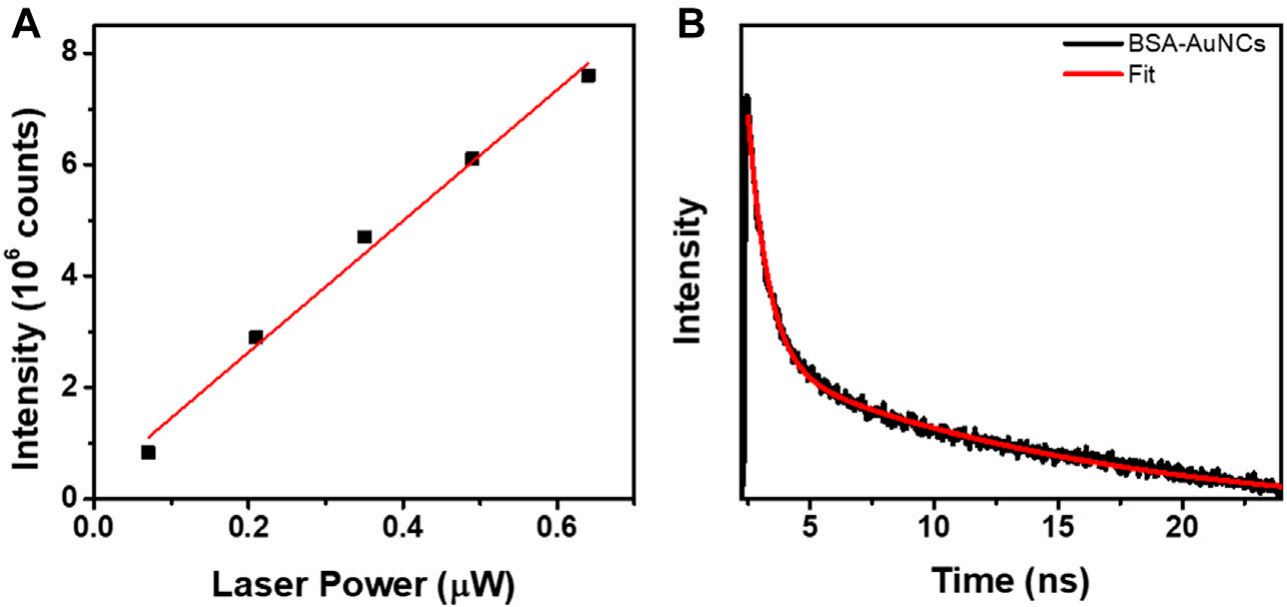
**(A)** The linear dependency of the OPE PL intensity exhibited by the entire BSA-AuNCs microflake on the excitation laser power (0.07–0.67 µW, R2 = 0.988). **(B)** OPE lifetime decay corresponding to BSA-AuNCs microflake under an excitation of 405 nm (0.67 µW). The intensity of the lifetime decay is presented in a logarithmic scale.

BSA-AuNCs in powder state exhibit uniform and intense PL under the used excitation wavelength, while the intensity of the emission seems to be strongly correlated with the excitation laser power. Notably, after repeated excitations of the same microflake with different laser powers, the BSA-AuNCs show no trace of photobleaching, outcome that might not be able to achieve with a normal fluorophore (Zheng and Blanchard, 2013). The overall intensity of each FLIM image at different laser powers was extracted and presented in Figure 3A.

The PL of BSA-AuNCs microflake increases linearly with the increase of laser power, an expected phenomenon taking in consideration that at OPE the fluorescence emission intensity linear dependency can de described by the following equation (Variables that Influence Fluorescence Measurements, 2021):

$$I=2.303K^{\prime }\varepsilon bcP_{0},$$ (2)

where I is the fluorescence intensity, K’ is a constant that depends on the fluorescence quantum yield, the molecule’s geometry and other factors, ɛ is the molar absorptivity, b is the laser path length inside the molecule, c is the concentration of the molecule, and $P_{0}$ is the excitation laser power.

Moreover, a representative fluorescence lifetime decay curve was extracted from the recorded OPE FLM images, using the same 405 nm excitation, in order to investigate the lifetime of the BSA-AuNCs powder microflake’s PL. The decay curve extracted from the microflake, presented in Figure 3B along with the fitting curve, reveals a two-exponential behavior. Each calculated lifetime component, presented in Table 1 along with the average obtained value, was successfully assigned to a specific transition between the energy levels of BSA-AuNCs, under a pulse laser excitation.

**TABLE 1:** The PL lifetime decay parameters obtained for a BSA-AuNCs microflake powder under OPE and TPE.

Probe BSA-AuNCs	τ_1_ (ns)	A_1_ (%)	τ_2_ (ns)	A_2_ (%)	τ_av_ (ns)	χ^2^
Powder under OPE	13.1 ± 0.39	33.1	0.69 ± 0.02	66.9	12.1	1.27
Powder under TPE	6.8 ± 0.28	41.6	0.61 ± 0.01	58.4	6.1	1.12
Agarose-Phantom under TPE	6.7 ± 0.23	27.2	0.65 ± 0.01	72.8	5.5	1.02

**SCHEME 1:**
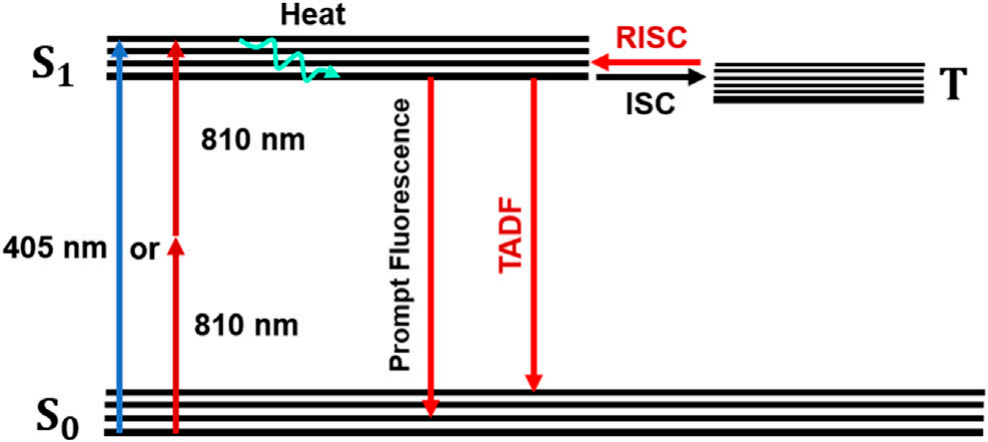
Jablonski diagram describing the PL emission of BSA-AuNCs.

Succinctly, when BSA-AuNCs absorb light (Scheme 1), electrons will transition mainly to the first excited singlet level (S1). After an extremely short period of time, electrons relax to the ground vibrational state of S1 where two processes can occur: intersystem crossing (ISC) or prompt fluorescence emission (PF). If the ISC process takes place, the electron’s spin multiplicity changes to the first excited triplet level (T). At this point, they can bounce back to S1 as a result of a thermal energy absorption, process called reverse-ISC (RISC). In the end, the electrons will relax through a temperature activated radiative process, also known as delayed fluorescence (TADF) (Wen et al., 2012). Consequently, the slower lifetime (**τ**_**1**_) can be assigned to ISC, while the faster component (**τ**_**2**_) corresponds to PF process. Unfortunately, the TADF lifetime (Yuan et al., 2013) is over the limit value that our equipment can measure. These results are in a good agreement with our previous reported data obtained in solution (Hada et al., 2021b).

**FIGURE 4:**
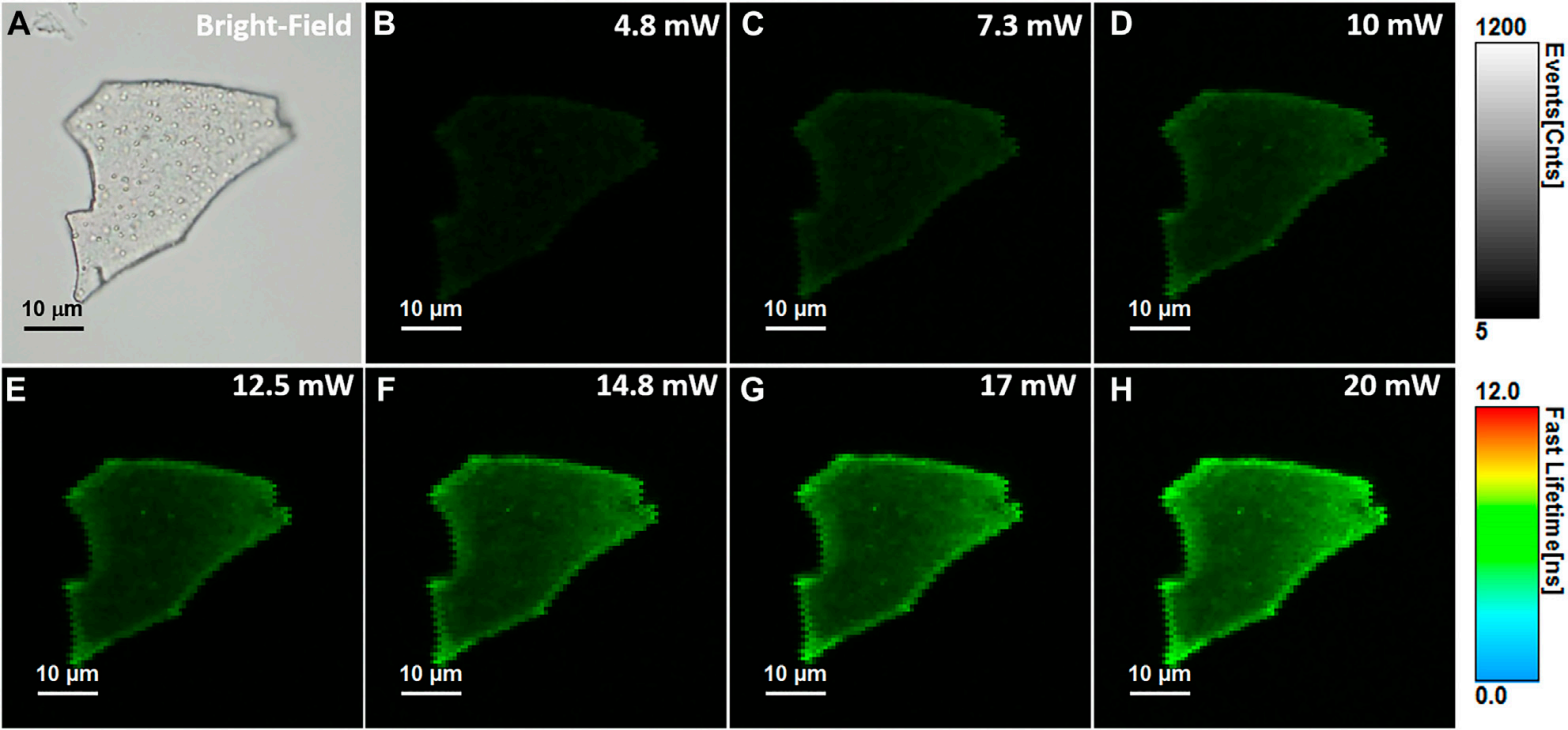
**(A)** Optical image of the investigated BSA-AuNCs microflake powder. **(B–H)** TPE FLIM images of BSA-AuNCs microflake powder obtained under 810 nm excitation and different laser power ranging from 5 to 20 mW at the exit of the laser cavity. All FLIM image are presented to the same intensity (0-1,200 counts) and lifetime (0–12 ns) scales. The images were recorded at a resolution of 128 × 128 px with an imaging speed of 49 s.

Compared to OPE methods, the TPE fluorescence imaging microscopy presents numerous advantages such as highly localized excitation providing background free information, elimination of out-of-focus absorption, reduced photobleaching and phototoxicity, the possibility to eliminate the pinhole, flexible detection geometries and more efficient photon detection, while maintaining a high image contrast (Benninger and Piston, 2013). Nonetheless, the TPE method requires longer wavelength photons, around the NIR region, giving the technique increased imaging depth and therefore the possibility to be applied in biomedical applications such as live cell and tissue imaging. Despite its superiority, the TPE method has only been recently exploited in the investigation of NCs’ PL (Bertorelle et al., 2018; Vangara et al., 2018; Olesiak-Banska et al., 2019), but up to our knowledge none of them on NCs in solid-state. Therefore, we performed here TPE FLIM assays on the same BSA-AuNCs microflake under an 810 nm excitation using different excitation power (ranging between 5 and 20 mW at the laser cavity exit). The obtained TPE FLIM images together with the corresponding optical image of the microflake are presented in Figure 4.

**FIGURE 5:**
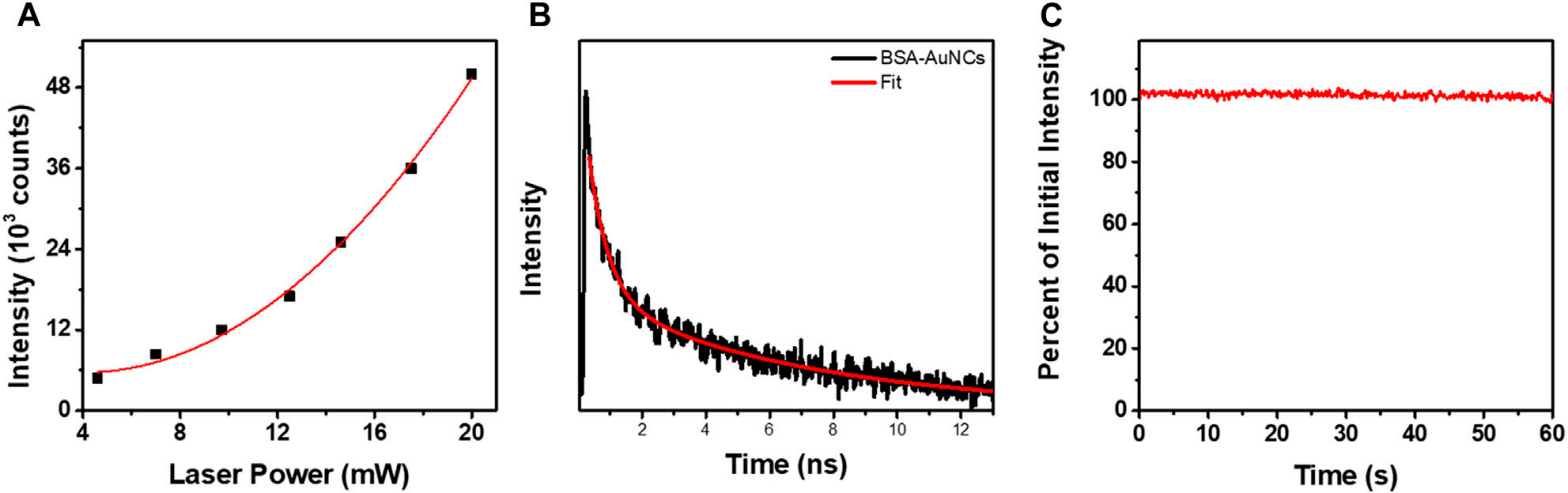
**(A)** Quadratic dependency of the TPE PL intensity of entire BSA-AuNCs microflake powder on the laser power (5–20 mW). The **(B)** TPE lifetime decay obtained for BSA-AuNCs microflake powder at an excitation of 810 nm (5 mW). The intensity of the lifetime decay is presented in a logarithmic scale. **(C)** The photostability of BSA-AuNCs microflake powder under continuous pulsed excitation at 810 nm for 1 min.

Remarkably, the BSA-AuNCs microflake exhibits an intense and relatively uniform fluorescence emission under TPE at 810 nm. As it is well known that under NIR excitation, water and chromophores exhibit minimal scattering and absorption, these results prove the ability of BSA-AuNCs to be explored as potential fluorescence contrast agents in deep tissue imaging. Moreover, the quadratic dependence of their PL’s intensity (overall intensity of each FLIM image) on the excitation laser power (Figure 5A) certainly demonstrates that the obtained emission originates from a TPE process.

The fitting procedures performed on the lifetime decay curve extracted from the BSA-AuNCs microflake (Figure 5B) exposes the two previously described processes: PF (**τ**_**2**_ **)** and ISC (**τ**_**1**_), demonstrating that the PL properties of the AuNCs preserved under TPE. In our previous work (Hada et al., 2021b), we observed that by increasing the excitation wavelengths the emission red-shifts, while the fluorescence lifetimes decrease, due to the increasing probability of the TADF process to take place. Therefore, we believe that when using 810 nm TPE, the TADF emission of BSA-AuNCs is favored, thus the slower fluorescence lifetime component decreases compared to the 405 nm OPE conditions (Table 1). Moreover, we need to take in consideration that when the BSA-AuNCs are analyzed under OPE, a significantly larger volume of sample is irradiated compared to TPE, where the irradiation is highly localized and can produce local heating higher than in the case of OPE. Therefore, the differences observed between OPE and TPE lifetimes can be related to the TADF process as multiple studies already demonstrated that the lifetime of TADF component decreases when local temperature increase occurs, while no significant effect on the PF lifetime is observed (Wen et al., 2012; Lin et al., 2017). Therefore, we assume that a local heating takes place when BSA-AuNCs are analyzed under TPE, which would result in a decrease of the TADF lifetime component. Even though our system does not allow the precise measurement of extremely long TADF lifetimes component, we were capable to record the **τ**_**1**_ lifetime component, which according to literature is assigned to the ISC process and represents a mandatory process which needs to take place in order for the TADF emission to happen. More importantly, the ISC process is extremely dependent on the sample temperature (Lim et al., 1966; Adolph and Williams, 1967; Widman and Huber, 1972), while its lifetime decreases with the temperature (Wen et al., 2012), an effect which we were able to detect through the lifetime variations from 13.1 ± 0.39 ns under OPE to 6.8 ± 0.28 ns under TPE. Furthermore, we observed in our previous work (Hada et al., 2021b) that BSA-AuNCs in solution present a temperature dependent emission. Particularly, as the temperature increases the BSA-AuNCs PL emission decreases in intensity, but also present a red shift, which is another indication that the TADF emission is favorized at higher temperatures. Noteworthy, the BSA-AuNCs PL lifetime components do not overlap with the lifetime of endogenous intracellular autofluorescence, typically between 2 and 3 ns. Finally, we investigated the photostability BSA-AuNCs microflake under continuous pulsed irradiation at 810 nm for 1 min. Figure 5C shows that, BSA-AuNCs kept more than 99.5% of their initial PL, an extremely important advantage when it comes to bioimaging applications which proves the feasibility of using them as reliable stable contrast agents for non-invasive imaging assays of biological samples using NIR irradiation.

### Two Photon Excitation Fluorescence Lifetime Imaging Microscopy Assays on Agarose-Phantom with Bovine Serum Albumin Stabilized AuNCs

**FIGURE 6:**
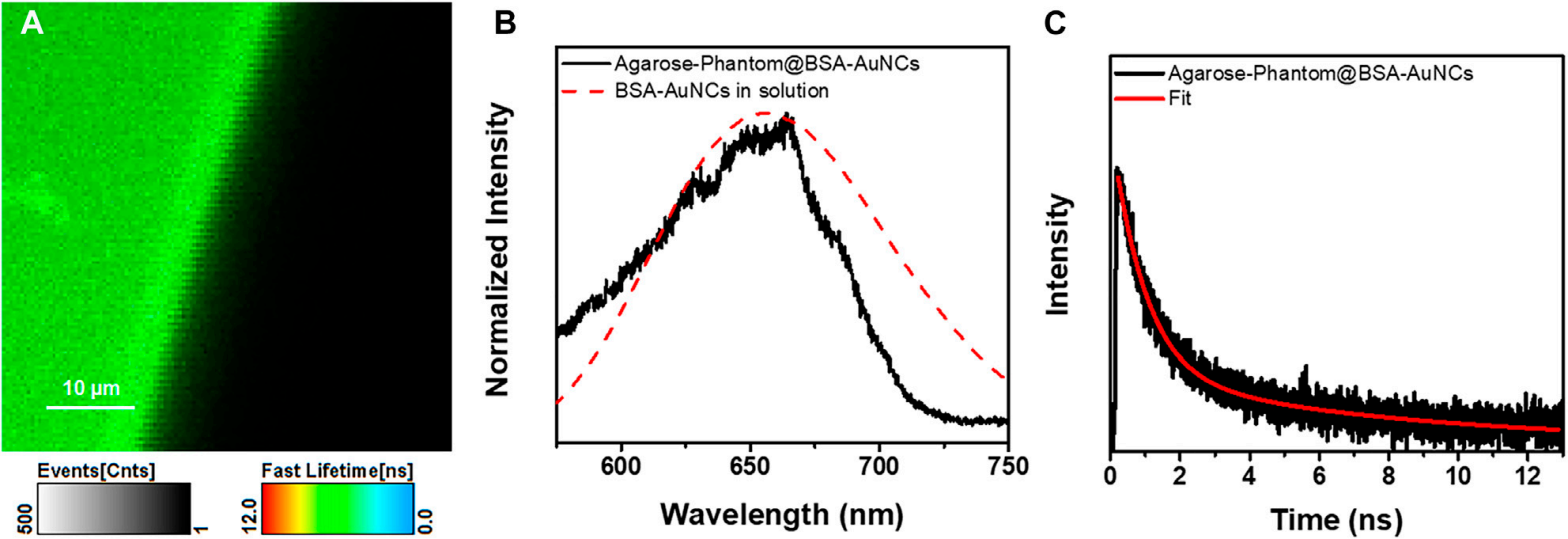
**(A)** TPE FLIM image (the image was recorded at a resolution of 200 × 200 px with an imaging speed of 105 s) and **(B)** the corresponding emission spectrum of the Agarose-Phantom@BSA-AuNCs (black line) obtained under excitation at 810 nm (20 mW) and of BSA-AuNCs (dashed red line) in solution at 405 nm excitation. **(C)** TPE lifetime decay curve obtained for Agarose-Phantom@BSA-AuNCs at 810 nm excitation (20 mW). The intensity of the lifetime decay is presented in a logarithmic scale.

Bionanomedicine is among the most exploited research topic, while its main goal is to come with solutions to medical problems using nanometric devices. However, multiple promising studies stop at the *in vitro* level. For example, in our previous work, we demonstrated the ability of BSA-AuNCs to perform as fluorescence contrast agents for the visualization of ovarian cancer cells under visible light excitation. However, it is well known that NIR excitation not only provides the possibility of deep tissue imaging but is also non-invasive. Therefore, we internalized the BSA-AuNCs inside a cancer tissue mimicking agarose-phantom (Agarose-Phantom@BSA-AuNCs) and tested their efficiency as contrast agents under non-invasive NIR TPE in simulated *ex vivo* environment, as a novel proof of concept of AuNCs-based NIR tissue imaging approach. The TPE FLIM image of Agarose-Phantom@BSA-AuNCs obtained under 810 nm excitation (Figure 6A) reveals an intense and homogenous fluorescence signal originating from the entrapped BSA-AuNCs. We point out that reference Agarose-Phantom exhibits unsignificant signal under the same conditions. In the obtained image, we are clearly able to differentiate between areas with BSA-AuNCs (green) and without (black), which demonstrates the ability of BSA-AuNCs to emit a strong PL under NIR excitation, when embedded in a cancer tissue-mimicking solid phantom. In addition, the spectral information together with PL lifetime values obtained for the Agarose-Phantom@BSA-AuNCs are in good agreement with the results obtained on lyophilized powder, proving that the emissive properties of BSA-AuNCs maintained even inside the tissue mimicking agarose phantom. Specifically, the PL spectrum extracted from Agarose-Phantom@BSA-AuNCs, under TPE at 810 nm, matches the PL of BSA-AuNCs in both solution and solid-state phase (Figure 6B).

Additionally, the PL lifetime components obtained after fitting the decay curve from Figure 6C, presented in Table 1, are similar to the values obtained for BSA-AuNCs powder under TPE, with a small variation of contributions, most probably due to different environmental parameters. Therefore, the aforementioned results confirm the uniform distribution and the excellent staining ability of BSA-AuNCs inside tissue-like phantoms, under TPE, demonstrating, as a proof of concept, the ability of BSA-AuNCs to perform as reliable fluorescent contrast agents in future *ex vivo* or even *in vivo* non-invasive NIR FLIM-based imaging applications.

## Conclusion

To conclude, we have successfully investigated for the first time the PL properties of solid-state BSA-AuNCs under OPE and TPE *via* FLIM measurements coupled with fluorescence spectroscopic investigations. Steady-state fluorescence spectroscopy measurements revealed that BSA-AuNCs, in both solution and powder state, feature a stable PL in the first biological window. Moreover, the absolute quantum yield of the BSA-AuNCs was calculated to be 6% under 405 nm excitation, which, up to our knowledge is the first time an absolute value is reported for BSA-AuNCs. Notably, a low degree of photobleaching was observed under both OPE and TPE continuous irradiation as the lyophilized BSA-AuNCs kept 99.5% of their initial PL under both excitation conditions. The FLIM assays performed on single powder microflakes, under both OPE and TPE, revealed a homogeneous PL, while fluorescence lifetime analysis confirmed the two possible emission processes (PF and DF) occurring in BSA-AuNCs. The quadratic behavior of the BSA-AuNCs PL intensity against the excitation power, validated its TPE origins. Finally, as a proof of concept, simulated *ex vivo* FLIM assays performed for the first time on tissue mimicking agarose phantom with BSA-AuNCs insertions, performed under TPE, demonstrated the high efficiency of BSA-AuNCs as fluorescence contrast agents. Taking into consideration the significant role that tissue-like phantoms play in the development and validation of new bioimaging technologies, our results prove that BSA-AuNCs represent promising candidates for *ex vivo* and *in vivo* non-invasive NIR tissue imaging.

## Data Availability

The original contributions presented in the study are included in the article/supplementary files, further inquiries can be directed to the corresponding author/s.
